# An Initial Audit of Delirium Detection and Management in an Intensive Care Setting

**DOI:** 10.1192/bjo.2024.539

**Published:** 2024-08-01

**Authors:** Yzobelle Barcelos, Kirthika Mohanathass

**Affiliations:** East and North Hertfordshire NHS Trust, Stevenage, United Kingdom

## Abstract

**Aims:**

In the intensive care unit (ICU), delirium occurs in up to 80% of patients on mechanical ventilation. Delirium is associated with an increased risk of morbidity and mortality, long-term cognitive decline, and risk of reintubation. This initial audit aims to identify areas of improvement in the early detection, prevention, and management of delirium in the ICU of the general hospital following trust guidelines.

**Methods:**

In this baseline audit, data was collected about all inpatients on admission over a 7-week period (81 patients in total). The parameters audited were in accordance with trust guidance on the management of delirium and compliance to this was recorded. Parameters included: the correct use and documentation of screening tools, type and cause of delirium, pharmacological and non-pharmacological management, and other demographics such as sensory impairment and length of stay. Confused patients handed over verbally during ward rounds were also assessed again at the time, with documentation and parameters reviewed.

**Results:**

Of the 81 inpatients in the ICU, 20 were observed with delirium during their stay. The documentation of delirium via the CAM-ICU screening tool was incorrect in 25% of patients with delirium (PWDs). Furthermore, behaviour (including sleep) was only monitored for 15% of PWDs and 0% had a complete “This is me” document (support tool for patient-centred care).

Sensory aids were not available for 50% of PWDs and 25% of this group had drug/alcohol dependence. A diagnosis of delirium was only formally documented in 40% of PWDs and of these, 15% had the type of delirium documented. Only 8 PWDs received a specific management plan, with 6 PWDs receiving haloperidol or lorazepam for agitation. Non-pharmacological managements were not documented.

The average length of stay in the hospital was 20% longer in PWDs compared with non-delirium patients, with 10 deaths in the ICU; 50% of these being PWDs.

**Conclusion:**

There is a lack of accurate documentation and a lack of medical optimisation for PWDs, which may lead to missed delirium diagnosis, greater risk of mortality and longer hospital stays. The results highlight a need for further education about delirium in the ICU, to increase awareness for better detection, prevention and promotion of appropriate delirium management and formal documentation as per trust guidelines. Furthermore, a need to consider alternative pharmacological management for delirium, specifically in the ICU where lorazepam and haloperidol may not be suitable in consideration of anaesthetic drug interactions and respiratory support requirements.

